# AGR2: a secreted protein worthy of attention in diagnosis and treatment of breast cancer

**DOI:** 10.3389/fonc.2023.1195885

**Published:** 2023-05-01

**Authors:** Ke Zhang, Yuan Li, Xiangyi Kong, Chuqi Lei, Huaiyu Yang, Nianchang Wang, Zhongzhao Wang, Hu Chang, Lixue Xuan

**Affiliations:** ^1^ Department of Breast Surgical Oncology, National Cancer Center/National Clinical Research Center for Cancer/Cancer Hospital, Chinese Academy of Medical Sciences and Peking Union Medical College, Beijing, China; ^2^ Department of Cancer Prevention, National Cancer Center/National Clinical Research Center for Cancer/Cancer Hospital, Chinese Academy of Medical Sciences and Peking Union Medical College, Beijing, China; ^3^ Administration Office, National Cancer Center/National Clinical Research Center for Cancer/Cancer Hospital, Chinese Academy of Medical Sciences and Peking Union Medical College, Beijing, China

**Keywords:** AGR2, breast cancer, protein disulfide isomerase, protein-protein interaction, protein secretion

## Abstract

AGR2 is a secreted protein widely existing in breast. In precancerous lesions, primary tumors and metastatic tumors, the expression of AGR2 is increased, which has aroused our interest. This review introduces the gene and protein structure of AGR2. Its endoplasmic reticulum retention sequence, protein disulfide isomerase active site and multiple protein binding sequences endow AGR2 with diverse functions inside and outside breast cancer cells. This review also enumerates the role of AGR2 in the progress and prognosis of breast cancer, and emphasizes that AGR2 can be a promising biomarker and a target for immunotherapy of breast cancer, providing new ideas for early diagnosis and treatment of breast cancer.

## Introduction

In 1998, Devon A.Thomson and Ronald J.Weigel of Stanford University in the United States used suppression subtractive hybridization (SSH) to screen the homologous gene with Xenopus anterior gradient-2 (XAG-2) from the cDNA library of estrogen receptor positive breast cancer cell line MCF7, named hAG-2, or anterior gradient-2 (AGR2) (1). AGR2 was only expressed in estrogen receptor positive breast cancer cell lines, but not in estrogen receptor negative breast cancer cell lines, which attracted great attention when it was found. Subsequently, a large number of studies showed that AGR2 was overexpressed in more than half of cases in breast cancer, prostate cancer, pancreatic cancer, esophageal cancer, which had a certain correlation with the development stage and pathological characteristics of the tumor, and might be involved in the process of tumor cell metastasis, survival, invasion and so on, indicating that AGR2 may be a new key gene related to cancer regulation and biomarker. The regulatory effect and mechanism of AGR2 on tumor is an important hotspot in the field of tumor research. The correlation between AGR2 expression and ER positive rate of breast cancer cell lines and the ability of estradiol to induce its expression suggests that AGR2 may mediate the normal physiology and estrogen effect of breast cancer (2). This article focuses on the research progress of AGR2 and the occurrence, development and clinicopathological relationship of breast cancer.

## AGR2 gene

A clone containing AGR2 gene was isolated from human genomic DNA library (3). The whole clone was labeled with biotin and used as a probe for FISH analysis. Highly specific signals were detected in chromosome region 7p21.3 of all metaphase cells, indicating that AGR2 gene is located in the region of human chromosome 7p21.3. The AGR2 gene spans a region of 50 kb in genomic DNA, containing 8 exons and 7 introns, and is mainly expressed in organs from endoderm (4). Hrstka et al. (5) carried out chromatin immunoprecipitation assay on AGR2 promoters. Compared with the control group, the amount of AGR2 promoter that coimmunoprecipitated with ERα antibody was approximately twofold increased, indicating that the transcription of AGR2 is estrogen responsive at the molecular level.

## Structure of AGR2 protein

**Figure 1:**
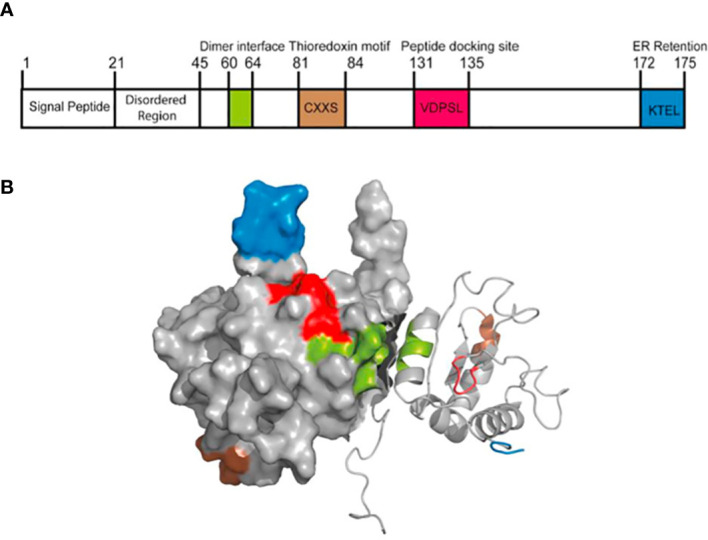
The structure of AGR2 protein. **(A)** The primary structure of AGR2 protein. The function of human AGR2 is highlighted; From left to right are signal peptide, N-terminal internal disordered region, dimerization motif (green), thioredoxin motif (brown), peptide docking site (red) and ER retention motif (blue). The structural domains of AGR2 determine its diverse functions. **(B)** Solution structure of dimer AGR2. The color coding of the functional pattern is based on **(A)**. Reprinted from (9). © 2020 The Author(s).

AGR2 protein, an endoplasmic reticulum resident protein mainly expressed in human epithelial cells, is composed of 175 amino acid sequences with a relative molecular weight of 20000 (6). It was assigned to the human protein disulfide isomerase (PDI) family later than all other members. Mainly located in the endoplasmic reticulum, PDI family proteins have 1-4 active motifs CXXC, which can catalyze the formation and isomerization of protein disulfide bonds, so as to stabilize proteins, and can also be used as molecular chaperone to inhibit protein aggregation (7). Park et al. (8) found that AGR2 is important for the production of intestinal mucin 2 (MUC2) *in vivo* by studying mouse intestinal epithelial cells. MUC2 is a cysteine-rich glycoprotein, which forms a protective mucus gel in the intestine. A cysteine residue in AGR2 thioredoxin-like domain forms mixed disulfide bonds with MUC2, which is a prerequisite for MUC2 secretion by intestinal epithelial cells. This indicates that AGR2 has two key properties of PDI: endoplasmic reticulum localization and functional thioredoxin-like domain (**Figure 1**).

Most protein-protein interactions in mammals are driven by linear peptide motifs. Similarly, three classical linear motifs in AGR2 define its core biochemical determinants (10). First, the N-terminal hydrophobic sequence (amino acids 1-20) directs AGR2 into the endoplasmic reticulum, which contains a secretory signal sequence that can be cutted, and the cutting site is located between Ala20 and Lys21. Secondly, the C-terminus includes another classical linear peptide motif, the endoplasmic reticulum retention motif containing the tetrapeptide sequence of lysine (K), threonine (T), glutamate (E), leucine (L), abbreviated as KTEL. This sequence is conserved in all vertebrates from Xenopus laevis to human (11). KTEL motif can bind to three known KDEL receptors, leading to ER localization (12). KTEL motif has specific functions. Gupta et al. (11) used two different cell lines, in which AGR2 induced the expression of EGF receptor ligand amphibian glycoprotein or transcription factor CDX2, and found that only the highly conserved wild-type carboxy terminal KTEL motifs could produce appropriate results. Deletion of KTEL motif will lead to AGR2 secretion and AGR2 loss of function. When the carboxyl terminal KDEL or KSEL is used instead of KTEL, the AGR2 function will also be lost. However, compared with the classical KDEL sequence, the affinity between KTEL motif and KDEL receptor is lower. This may have a significant impact on the transport of AGR2 protein to different compartments in cells and its corresponding functions. AGR2 can escape the ER localization mechanism and be secreted to play an autocrine/paracrine role (13). Dumartin et al. (14) found that AGR2 was also located on the outer surface of pancreatic cancer cells expressing AGR2. At the same time, AGR2 can also exist in the extracellular space, serum and urine (15, 16), which opens up other ways for its role in the tumor microenvironment. In order to explore the functional significance of KTEL motif on AGR2 secretion, Fessart et al. (17) used HEK-293T cells that did not secrete AGR2 and used different AGR2 mutation structures, in which KTEL motif was mutated into KDEL, K172D, K172A or stop was inserted before KTEL (ΔKTEL). The results showed that the level of extracellular AGR2 in mutant cells was at least equal to that observed in wild-type cells. This suggests that the secretion of AGR2 protein may be independent of its KTEL motif. In conclusion, KTEL motif is more likely to be necessary for intracellular AGR2 to function. In addition, the KTEL motif in AGR2 also plays a functional role in the metastasis pathway of cancer cells. Intracellular AGR2 can promote colorectal metastasis through KDEL-KDEL receptor-Gs-PKA axis (18).

The third key linear motif of AGR2 is CXXS motif. Classical thioredoxin has a conserved thioredoxin fold, which is composed of CXXC motif. CXXC motif mediates the formation of covalent bonds with downstream proteins containing cysteine, and then is resolved by oxidation-reduction (19). In contrast, AGR2 is part of the thioredoxin superfamily, which contains CXXS motif and lacks the ability of the dicysteine redox system to mediate the redox of downstream proteins (6). Therefore, it may block the antioxidant electron transfer system, thus creating a superoxide environment and enhancing the maintenance of cell damage during tumor formation (20). It contains a central and unique cysteine residue, through which, it is thought to mediate a non-redundant reaction when binding to the substrate (10). At the same time, this motif may form mixed disulfides with mucin (MUC1 (8), MUC2 (21) and MUC5AC (22)), contributing to their secretion. Nevertheless, the analytical nuclear magnetic resonance (NMR) of AGR2 structure shows that AGR2 can be stable in an antiparallel way through the Glu60-Lys64 interface and acts as a homodimer to catalyze the CXXS motif away from the interface (23). Therefore, compared with the typical thioredoxin motif, the dimer structure can be regarded as having the same stoichiometric redox capacity (9). Surprisingly, the deletion of 40 amino acids at the N-terminal of AGR2 could improve the stability of the dimer by three orders of magnitude (10). This suggests that the full-length AGR2 may tend to exist as a monomer rather than a complete dimer (24). The N-terminal plays a natural negative regulatory role to reduce the affinity of dimer. Therefore, pharmacological operation on the stability of AGR2 dimer is possible, because synthetic peptides from the N-terminal disordered region can reverse regulate the stability of AGR2 dimer, which suggests that we can develop a drug precursor that can change the stability of AGR2 dimer. Patel et al. (23) found that AGR221-175 with 20 amino acids removed from the N-terminal could significantly improve the adhesion rate of rat breast tumor cells. In contrast, AGR241-175 lacked the 21-40 region, which not only failed to improve the cell adhesion rate, but also showed a significantly reduced rate. The cell adhesion rates of monomer mutant protein E60A AGR221-175 and E60A AGR241-175 (48.3 ± 4.3%, 11.5 ± 0.3%) were not significantly different from the corresponding natural dimer protein AGR221-175 and AGR241-175 (P = 0.58, P = 0.74). This indicates that monomer and dimer forms have similar cell adhesion properties. Although the N-terminal 21-40 amino acids are disordered, they are specific in the role of cell adhesion. In addition to the dimer structure mediated by amino acids 60-64, AGR2 can also form disulfide bond via Cysteine-81 and reorient the dimer to different conformations (25). This homodimer triggers the activation of unfolded protein reaction (UPR) signaling pathway through the interaction with BiP/GRP78, and reduces ER stress-induced cell death.

## AGR2 and interaction partners

Protein-protein interaction (PPI) is important for the correct structure and function of most protein complexes (26). Protein-protein interaction may significantly contribute to the regulation of key biological processes, such as cell growth, proliferation and cell homeostasis (27). Therefore, the identification and analysis of the physical interactions between various proteins is essential for revealing the functions of physiological proteins and understanding the molecular mechanisms leading to human diseases. Existing data show that AGR2 can bind to a variety of proteins, such as nuclear protein, cytoplasmic protein and plasma membrane protein (28), as described in **Table 1**.

**Table 1:** List of AGR2 interacting proteins validated by protein-protein interaction assays.

Protein Name	Method	Interacting Domain	Influence	Reference
Endoplasmic reticulum				
TMED2	Endoplasmic reticulum mammalian protein-protein interaction trap (ERMIT), coimmunoprecipitation	Amino acid K66 and amino acid Y111	Control AGR2 dimerization	(29)
KDELR	Bimolecular fluorescence completion	KTEL motif (Amino acid 172 - 175)	Identification of three human KDEL receptors with different specificities	(12)
Plasma membrane				
DAG1	The yeast two-hybrid system	–	Cancer metastasis	(30)
EGFR	Protein immunoblots	–	EGFR can be transported to the cell surface, thus affecting the cell signal transduction	(31)
LYPD3(C4.4A)	The yeast two-hybrid system	–	Cancer metastasis	(30, 32)
EpCAM	ELISA, colocalization, proximity ligation assay	The structural ring of amino acids 131 – 135 (VDPSL)	Use linear peptide motif as a tool for discovering new protein-protein interactions	(24)
MUC1	coimmunoprecipitation experiments	–	Initiation and progress of carcinogenesis	(21)
MUC2	coimmunoprecipitation experiments	CXXS motif	Without AGR2, mice could not produce intestinal mucin	(8)
Immature MUC5AC and MUC5B	coimmunoprecipitation experiments	–	excessive mucus production caused by allergic airway inflammation	(22)
Cathepsin B (CTSB) and D (CTSD)	Two-dimensional fluorescence difference gel electrophoresis (2D-DIGE)	–	Cancer metastasis	(14)
Cytoplasm				
Reptin	The yeast two-hybrid system	Amino acids 104 - 111	Cancer cell growth	(33)
TSG101	stable-isotope labelling by amino acids in cell culture (SILAC) analysis	–	P53 inhibitor, leading to tumor transformation	(34)
Ki67	SILAC analysis	–	Promote cell proliferation	(34)
TAK1/TAB1/2 complex	tandem affinity purification combined with liquid chromatography tandem mass spectrometry (TAP-LC-MS/MS)	–	Promote tumor metastasis	(35)
RASSF5/STK3/4	TAP-LC-MS/MS	–	Consolidate the ability to inhibit apoptosis	(35)
Mitochondria				
UNG1	Immunoprecipitation	–	Stabilize UNG1 and enhance its enzyme activity in DNA repair	(36)

Fletcher et al. (30) found two proteins interacting with AGR2 by yeast two-hybrid system, GPI anchored C4.4a protein and DAG-1 protein. Their expression in breast cancer samples was higher than that in adjacent normal tissues, and the protein expression in ER positive breast cancer was higher than that in ER negative breast cancer. C4.4a protein can bind to its ligand’s laminin 1 and 5, and is associated with galectin 3 to promote cell metastasis (37). This associates AGR2 with GPI-anchored receptor proteins involved in hormone reactivity, cell adhesion, migration and metastasis. AGR2 may promote tumor metastasis through receptor adhesion and functional regulation. At the same time, the interaction with C4.4a and DAG-1 proteins may be a feasible target for the intervention of estrogen responsive breast cancer, which promotes researchers’ interest in the research of proteins interacting with AGR2. Although C4.4a and DAG-1 proteins have not been biologically verified as real protein-protein interactions in human cells, Kumar et al. (38) found the interaction between newt extracellular receptor Prod1 and newt AGR2 using yeast two-hybrid system, and verified the direct signal transduction role of AGR2 in amphibian limb regeneration. Human CD59 protein and newt Prod1 protein have 23% homology, and both belong to Ly6 superfamily with the same core motif CCXXXXCN (39). Therefore, it is speculated that CD59 may also be a binding target of AGR2 in human and needs to be validated in human cells in the future.

The most well characterized AGR2 binding protein is the AAA+ superfamily protein Reptin (33). AGR2 interacts with Reptin by forming a dispersed octapeptide loop domain through its 104-111 amino acid residues, which is a stable complex to regulate the ATPase activity and helicase function of Reptin. At the same time, this study showed that Reptin could be overexpressed in human breast cancer. Considering that the mutation of ATP binding site of Reptin will affect its oligomerization level, thermal stability and stability of binding with AGR2, the modification of ATP binding site is of great significance to explore the role of Reptin-AGR2 complex in the growth of breast cancer cells.

AGR2 protein can specifically bind to a specific peptide motif (TTIYY), thus driving the interaction with other proteins (40). This motif is rich in membrane associated protein, which indicates that AGR2 plays a role in this kind of protein. Mohtar et al. (24) located the dominant region of interaction with TTIYY peptide in AGR2 by hydrogen deuterium exchange mass spectrometry. It is in the structural ring of amino acids 131-135 (VDPSL). This intrinsic sequence specific peptide binding activity in AGR2 is important for its carcinogenic function, which has been used to mine human protein databases to search for proteins with similar motif. If we can find a protein corresponding to the AGR2 specific peptide motif, which can compete with the client protein in the carcinogenic pathway to bind AGR2, it will help to weaken the role of AGR2 in cancer growth and metastasis.

In addition, under normal conditions, AGR2 has been shown to interact with subtype of uracil DNA glycosylase protein (UNG1), which plays a key role in base excision and repair of mitochondria (36). Guo et al. (41) observed that extracellular AGR2 directly interacted with vascular endothelial growth factor (VEGF) and fibroblast growth factor 2 (FGF2) and enhanced their effects, contributing to angiogenesis and tumor growth. In addition, AGR2 may also induce the expression of lactate dehydrogenase A (LDHA), phosphoglycerate kinase 1 (PGK1), kallikrein 2 (HK2) and enolase 1α (ENO1) through the MUC1/HIF-α pathway (42), thus induce glycolysis of cancer cells, promote cell proliferation, migration, invasion and tumor growth. AGR2 can target and regulate the coactivators of Hippo signaling pathway, YAP-1 and amphiregulin (AREG) (31). AREG may interact with epidermal growth factor receptor (EGFR) to promote the growth of cancer cells. In addition, AGR2 can also play a role as an inhibitor of p53 (20), making the p53-dependent cell proliferation checkpoint ineffective. AGR2 may inhibit the phosphorylation of p53 at Ser15 and Ser392 sites by targeting the plasma membrane, thereby preventing cell apoptosis. At the same time, AGR2 up-regulates dual specific phosphatase 10 (DUSP10) (43, 44), thereby inhibiting p38 mitogen activated protein kinase (p38 MAPK) and preventing the activation of p53. MAPK/ERK signaling pathway may also be involved in the role of AGR2 in tumor (45). Under physiological emergency conditions, AGR2 induced response in MDA-MB-231 cell line can be effectively blocked by PD98059, a specific inhibitor of ERK1/2. The purification of AGR2 binding protein by TAP also showed that the TAK1/TAB1/2 complex in MAPK signaling pathway might be involved in the process of AGR2 regulating tumorigenesis and metastasis (35). AGR2 also induces tumor metastasis by regulating mTOR complex 2 (mTORC2) pathway (46), promotes tumor cell dissemination through post transcriptional activation of cathepsin B (CTSB) and D (CTSD) (14), and prevents the activation of transforming growth factors β (TGF- β), which is involved in epithelial mesenchymal transition (EMT) during tumor invasion and metastasis, in order to maintain the epithelial phenotype (47). To summarize, intracellular and extracellular AGR2 can interact with other proteins through their intrinsic motifs and secretion functions, playing an essential role in tumor cell growth, angiogenesis, inhibition of apoptosis, and tissue metastasis.

## Expression and regulation of AGR2

AGR2 can participate in a variety of biological effects through protein-protein interactions. Meanwhile, AGR2 activity can also be regulated in a variety of ways. In breast cancer, AGR2 co exists with estrogen receptor and is induced by estrogen (2). Li et al. (48) deciphered that insulin-like growth factor-1 (IGF-1) significantly induced AGR2 in MCF7 cell line through the estrogen response element (ERE) between -802 and -808 bp and the leucine zipper transcription factor binding site between -972 and -982 bp on the AGR2 promoter. Knockdown of AGR2 can reduce IGF-1-induced cell proliferation, migration and cell cycle progression, which indicates that AGR2 is a key regulator involved in the development of IGF-1-induced breast cancer. However, the expression of AGR2 is not entirely dependent on estrogen. Other factors can also induce its expression. A study (45) showed that under hypoxia or serum-free conditions, the expression of AGR2 in ER negative breast cancer cell line BT20 was 8 times higher than that in normal condition after 24 hours of culture. The expression of estrogen receptor in MDA-MB-231 cell line was analyzed in parallel with the induction level of AGR2. It was found that AGR2 was also expressed when estrogen receptor was not induced. The possible mechanism is endoplasmic reticulum stress in extreme environment. Cells activate a series of complementary adaptive mechanisms to respond to the increased demand for protein folding in. This adaptive mechanism is called unfolded protein response (UPR) (49). UPR may regulate AGR2 through IRE1α and ATF6, which indicates the functional role of AGR2 in endoplasmic reticulum protein balance (50). At the same time, Jung et al. (51) found that Twist1 directly stimulated the activity of AGR2 promoter, which was necessary to induce the expression of AGR2 under hypoxia, indicating that AGR2 was a downstream effector protein of Twist1 to induce the growth and metastasis of breast cancer. Independent of estrogen, Ondrouskova et al. (52) found that HER2 can also up-regulate AGR2 by activating the extracellular signal regulated kinase 1/2 (ERK1/2) - Akt pathway, leading to the proliferation of breast cancer cells, indicating that in estrogen receptor negative breast cancer, AGR2 expression level is significantly correlated with HER2 expression status. hnRNPL is a protein that increases in metastatic lesions in breast cancer cells. Xiu et al. (53) found that hnRNPL-LINC02273 complex can recruit to the AGR2 promoter region, and epigenetically up-regulate AGR2 by enhancing local H3K4me3 and H3K27ac levels, activating AGR2 transcription and promoting cancer metastasis. The expression of AGR2 is also regulated by a variety of miRNAs. For example, MiR-135b-5p enhances the sensitivity of breast cancer cells to adriamycin by targeting AGR2 (54). Circular RNA CircPVT1 mediates AGR2-HIF-1α axis through MiR-29a-3p, promoting the growth, invasion, migration and inhibiting apoptosis of breast cancer cells (55). LncRNA AFAP1-AS1 induces drug resistance via miR-653-5p/AGR2 axis (56). Therefore, clarifying the upstream and downstream proteins involved in AGR2 interactions and regulating the activity of AGR2 proteins by intervening in related molecular pathways is a strategy for inhibiting cancer growth and metastasis.

**Figure 2:**
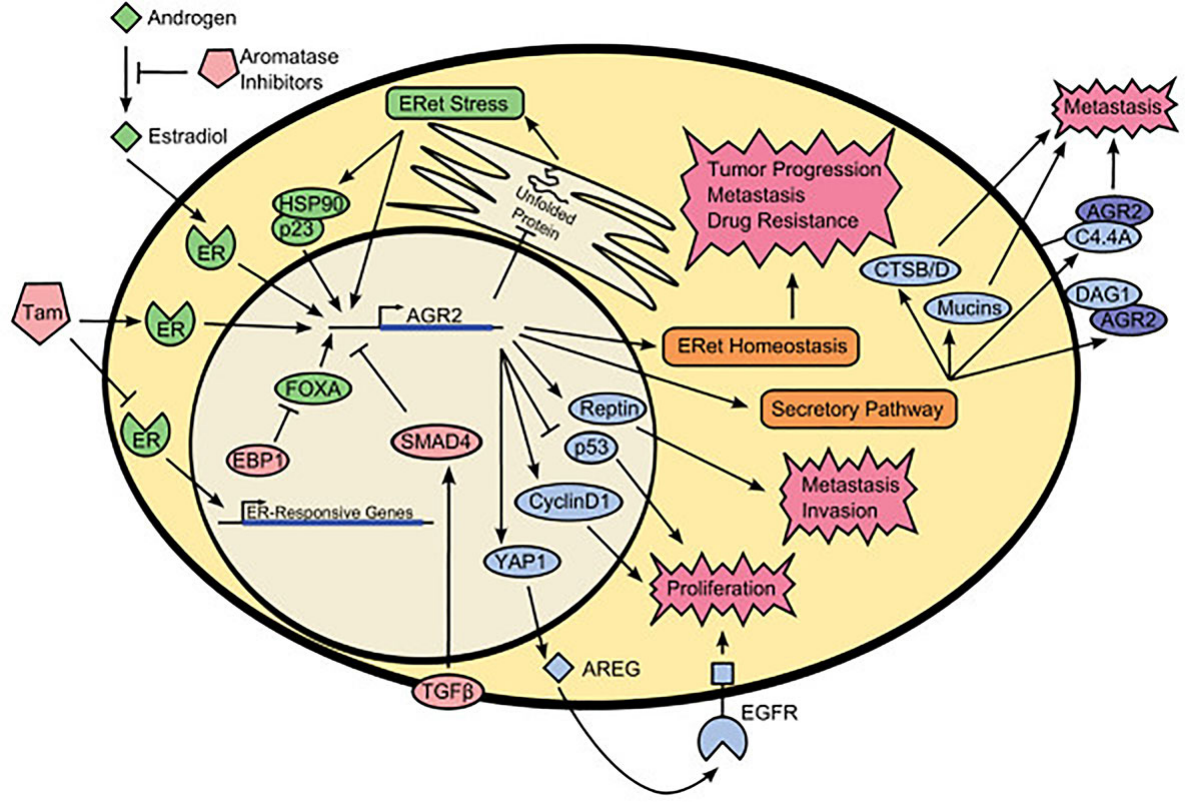
Schematic representation of AGR2 interactome. The green colored portion is the upstream activators of AGR2. The red colored portion is the upstream inhibitors of AGR2. The blue colored portion is proteins that interact with AGR2. AGR2 interacts with different proteins in the nucleus, cytoplasmic matrix, cell membrane, and outside of the cells to promote protein trafficking, protein homeostasis, cell signaling and proliferation, tumor progression and metastasis, drug resistance. Understanding the cancer promoting mechanism of AGR2 protein can help us better formulate cancer treatment strategies. Tam, tamoxifen; EBP1, ErbB3-binding protein 1; TGF-β, transforming growth factor β; ER, estrogen receptor; ERet, endoplasmic reticulum; FOXA, forkhead box family members A1 and A2; HSP90, heat-shock protein 90; YAP1, yes-associated protein 1; AREG, amphiregulin; CTSB/D, cathepsin B and cathepsin D; C4.4A, LY6/PLAUR domain containing 3; DAG1, dystroglycan 1; Reprinted from (2). Copyright © 2013 BioMed Central Ltd.

In conclusion, AGR2 has a unique primary protein structure, including secretory signal, endoplasmic reticulum retention sequence, as well as protein disulfide isomerase active site and a variety of protein binding sequences, which endows AGR2 with diverse roles in breast cancer cells. Intracellular AGR2 can promote the growth and survival of cancer cells, while extracellular AGR2 can be defined as a microenvironment regulator that makes cancer cells more aggressive (57). Intracellular AGR2, as a protein disulfide isomerase, catalyzes the proper folding of multiple client proteins through PDI activity. For example, AGR2 can mediate the maturation of receptors including MUC5 and MUC2 by forming mixed disulfide bonds (8, 22). At the same time, AGR2 is very important in endoplasmic reticulum regulation and quality control. Overexpression of intracellular AGR2 may represent an intermediate entity between endoplasmic reticulum and tumor development (9). Endoplasmic reticulum stress and UPR activation can lead to the development of cancer (58). Under normal and basic condition, AGR2 mainly exists in homodimers. During endoplasmic reticulum stress, AGR2 dimers dissociate in a dose-dependent manner and form functional complexes with endoplasmic reticulum related degradation mechanism (ERAD) to isolate misfolded proteins from endoplasmic reticulum (29, 59). Conversely, if the balance between AGR2 dimer and monomer is broken, it will lead to the activation of pro-inflammatory response and the release of AGR2 into the extracellular environment (29), which indicates that the breaking of the relative balance between AGR2 dimer and monomer may be a sign of protein imbalance in endoplasmic reticulum (60). Although AGR2 usually exists in endoplasmic reticulum due to its protein folding and protein balance functions, AGR2 can escape the endoplasmic reticulum retrieval mechanism and locate in different cell compartments, such as cytoplasm, plasma membrane and extracellular environment, and affect downstream client proteins through protein-protein interaction. The secretion of extracellular AGR2 in cancer may be due to the saturation of endoplasmic reticulum receptor sites, because AGR2 is overexpressed in cancer cells (61). Clarke et al. (62) found that AGR2 is O-glycosylated when secreted from human and rat mammary epithelial cells, and the O-glycosylation of AGR2 may be important for AGR2-mediated cell adhesion. AGR2 in the extracellular environment may have a critical impact on the homeostasis of the tumor niche, which is a microenvironment conducive to tumor growth (28). Extracellular AGR2 can directly interact with vascular endothelial growth factor A through its thioredoxin motif, leading to enhanced VEGF/VEGFR2 signal transduction to promote vascular growth (63). Similarly, extracellular AGR2 can also directly promote the dimerization of VEGF and FGF2 and increase the concentration of active VEGF and FGF2 in the local environment of tumor, thus leading to the migration and aggregation of vascular endothelial cells and fibroblasts to the surrounding of tumor cells, and promoting angiogenesis, providing favorable conditions for the formation of tumor microenvironment (41). Meanwhile, AGR2 can be internalized into fibroblasts and cancer cells through endocytosis, then it will interact with β-catenin, resulting in β-catenin accumulation in the nucleus and regulating fibroblasts around tumor cells to affect tumor microenvironment (TME) (64, 65). Extracellular AGR2 and ER-α can interact to induce the expression of IGF-1, thereby promoting the proliferation, migration and epithelial-mesenchymal transition process in breast cancer cells and enhancing drug resistance (48). The existence of extracellular AGR2 can transform non tumor organs into tumor organs and enhance their growth by about 10 times, which is independent of its thioredoxin folding and endoplasmic reticulum retention motif (17). In this context, it is important to increase the understanding of the mechanisms and signals of AGR2 expression, localization and function. Similarly, future studies are needed to evaluate the complex coordination network of AGR2 cell function, because the change of AGR2 expression may affect the function of its interacting partners in different ways and damage the homeostasis and protein stability (**Figure 2**).

## The clinical association between AGR2 and breast cancer

AGR2 was found by comparing the protein differences between ER positive and negative breast cancer cells (1). AGR2 only expressed in ER positive breast cancer cell lines, such as MCF7, T-47D, BT-474 and ZR-75, but not in ER negative breast cancer cell line MDA-MB-231 (66), which attracted a lot of attention once it was found, as described in **Table 2**. Later, experiments *in vivo (* 75) and *in vitro (* 5) confirmed that the expression of AGR2 protein was indeed regulated by estrogen. Fletcher et al. (30) used tissue microarray to show that AGR2 was expressed in 83% (n = 48) breast cancer cases, which was significantly correlated with ER expression (P = 0.01) and negatively correlated with EGFR expression (P = 0.009). Liu et al. (67) subsequently confirmed that the expression of AGR2 mRNA in MCF-7 breast cancer cells increased by 7.3 ± 0.2 times in the presence of estrogen. Immunohistochemical analysis of human breast tumors (n = 44) revealed that there was a significant correlation between ERα positive and AGR2 expression. Meanwhile, their research showed AGR2 could induce metastatic phenotype *in vivo*. The injection of AGR2 transfected rat mammary gland cells (Rama 37) into the mammary fat pad of homologous rats could induce a high incidence of lung metastasis, but the incidence of primary tumors in the rat model did not increase, which indicated that the expression of AGR2 may be related to metastasis. However, they did not analyze the correlation with patient survival. In addition, in a group of 225 ER positive breast cancer patients treated with tamoxifen, the survival rate of patients with AGR2 positive in breast cancer cells was lower than that of patients with AGR2 negative (68). In contrast, 126 patients with ER negative breast cancer did not show this relationship. Fritzsche et al. (69) studied the expression of AGR2 in 155 cases of breast cancer samples at the mRNA and protein levels, and confirmed that there was a significant correlation between the expression of AGR2 and ER status, but they also found that the expression of AGR2 was positively correlated with low cell proliferation rate, low-grade tumors and negative lymph nodes, indicating that AGR2 was associated with good prognosis of breast cancer. Compared with the above two different research results, the reason for the difference may be that the samples selected in the study are all tumors after endocrine therapy, and the prognosis of patients has a certain change. For example, the anti-estrogen effect of tamoxifen may affect the expression of AGR2 and bias the experimental results. Therefore, Barraclough et al. (70) performed only surgical treatment in 315 patients with operable breast cancer without adjuvant therapy including hormone therapy, and monitored the expression of AGR2 protein and the survival rate of patients. The results showed that after 20 years of follow-up, only 26% of patients with AGR2 positive cancer survived, while the survival rate of patients with AGR2 negative cancer was 96%, and the median survival time was significantly different, 68 months and more than 216 months respectively (p<0.0001), indicating that the presence of AGR2 in primary tumors is a possible prognostic indicator of poor prognosis in patients with breast cancer. Phoebe et al. (71) analyzed the main tumor mRNA data of women in the Molecular Taxonomy of Breast Cancer International Consortium (METABRIC) to determine AGR2 expression and disease-specific survival. The results showed that increased tumor AGR2 mRNA expression was associated with decreased disease specific survival (DSS) among 1341 women (P = 0.03). Vanderlaag et al. (76) knocked out AGR2 in breast cancer cell lines using siRNA technology, which not only inhibited cell growth, but also led to cell death, and reduced the expression of survivin and c-Myc in ER positive cell lines. Survivin and c-Myc are related to the metastasis and invasion of breast cancer. Kereh et al. (72) compared the expression of AGR2 in metastatic patients and non-metastatic patients by counting the expression of AGR2 antibody on ELISA through cross-sectional observation study, and found that the average value of metastatic AGR2 was significantly higher than that of non-metastatic patients, 3.77 ng/dl and 1.76 ng/dl respectively (P <0.01), which also confirmed that AGR2 expression was associated with breast cancer metastasis. Maarouf et al. (73) also used ELISA method to evaluate the concentration of AGR2 in serum samples of breast cancer patients and healthy controls with or without metastasis. The results showed that the average value of AGR2 in healthy control group was 2.93 ± 0.42 ng/ml (n = 56), that in breast cancer group was 5.62 ± 0.87 ng/ml (n = 118), and that in breast cancer metastasis group was 13.7 ± 3.2 ng/ml (n = 23). AGR2 in patients with metastatic breast cancer was significantly higher than that in healthy controls (p<0.0001). These studies showed that in patients with ER positive breast cancer, AGR2 significantly promoted the metastasis and invasion of breast cancer cells, and was positively correlated with the poor prognosis of patients.

**Table 2:** Clinical research of AGR2 and breast cancer.

Researcher	Research Object	Research Type	Conclusion	Reference
Fletcher et al.	46 cDNA samples derived from breast tumor tissues	Retrospective study	Correlated with ER expression. Negatively correlated with EGFR expression.	(30)
Liu et al.	44 specimens from breast cancer patients	Retrospective study	Correlated with ER expression. Induced metastatic phenotype *in vivo*.	(67)
Innes et al.	225 patients with ER positive breast cancer treated with tamoxifen	Retrospective study	Associated with low survival rate in ER positive patients.	(68)
Fritzsche et al.	155 breast cancer patients	Retrospective study	Correlated with low cell proliferation rate, low grade tumor and negative lymph node.	(69)
Barraclough et al.	315 breast cancer patients who underwent surgery without adjuvant therapy	Longitudinal study	A possible prognostic indicator of poor prognosis in patients with breast cancer.	(70)
Phoebe et al.	Main tumor mRNA data of women in the Molecular Taxonomy of Breast Cancer International Consortium	Retrospective study	Correlated with low disease specific survival rate.	(71)
Kereh et al.	21 breast cancer patients	Cross-sectional observational study	Associated with breast cancer metastasis.	(72)
Maarouf et al.	118 breast cancer patients	Cross-sectional study	Promoted the metastasis and invasion of cancer cells. Correlated with the poor prognosis.	(73)
Lacambra et al.	504 breast cancer patients	Retrospective study	Significant difference of AGR2 expression rate in different molecular subtypes of breast cancer.	(74)

When considering molecular stratification, Lacambra et al. (74) retrospectively analyzed the immunohistochemical data of 504 breast cancer patients, and found that the expression rate of AGR2 in luminal A (n=226) was 50.4%, in luminal B (n=191) was 50.3%, in HER2-OE (n=40) was 35%, and in triple negative diseases was 4.3% (basal-like breast cancer, BLBC was 4.8%, unclassified was 3.8%). The positive rate of AGR2 in different molecular subtypes was significantly different (p<0.001). The expression of AGR2 was positively correlated with the expression of ER, PR and androgen receptor (AR), and negatively correlated with the expression of EGFR (p=0.002) and CK5/6 (p<0.001). These results further verified the data previously, showing that AGR2 was overexpressed in ER positive breast cancer. Interestingly, another research (77) revealed that the low expression of AGR2 is associated with the low overall survival of luminal A and the worst relapse free survival of basal-like breast cancer (BLBC). On the other hand, the high expression of AGR2 leads to worse overall survival and relapse free survival in luminal B patients and HER2 positive patients. A study (78) aimed to identify biomarkers of HER2 dependent breast cancer by proteomic methods found that AGR2 was overexpressed in more than 40% of HER2 positive breast cancer. The knockout of AGR2 resulted in enhanced invasion of MDA-MB435 cells. In the survival analysis of HER2 subgroup, it was found that in HER2 positive breast tumors, AGR2 expression was significantly increased at both mRNA and protein levels. In addition, in estrogen and progesterone receptor negative and HER2 positive cases, the increased expression of AGR2 was significantly correlated with the worse prognosis of patients (52, 79), indicating that AGR2 may be related to HER2 signal transduction.

So far, AGR2 participates in various tumor processes, such as differentiation, proliferation, migration, invasion and metastasis (80), and plays an important role in the progress and prognosis of breast cancer through its overexpression and non-canonical localizations. With the deepening of research, we found that the high expression of AGR2 marks the possible metastasis of breast cancer, which is one of the indicators of poor prognosis of breast cancer patients. However, its specific role in each molecular subtype of breast cancer has not yet been clarified. In different molecular subtypes, the level of AGR2 expression is related to the prognosis of patients, which should be further explored. Therefore, it is necessary to carry out more retrospective and prospective studies to clarify the molecular function and clinical role of AGR2, taking into account the heterogeneity and complexity of breast cancer molecules and the impact of breast cancer chemotherapy (**Table 2**).

## AGR2 and biomarker

AGR2, as a promising biomarker, has aroused great interest because of its increased expression pattern in precancerous lesions, primary tumors and metastatic tumors, which is used to detect the most common cancers (81). As a secretory molecule, extracellular AGR2 can be detected in several biological fluid, including serum, plasma and urine, so it is a promising non-invasive biomarker. Meta analysis of 20 studies including 3285 patients showed that the increased expression of AGR2 was associated with poor overall survival in patients with solid tumors, especially breast cancer (82). Compared with primary breast tumors, the expression of a novel long non coding RNA called LINC02273 in metastatic lesions was significantly increased. When LINC02273 is combined with AGR2, it can be used as an independent prognostic factor to predict overall survival in patients with breast cancer (53). Maarouf et al. (73) confirmed that AGR2 could be detected in the serum of untreated breast cancer patients, and the level of AGR2 in patients was significantly higher than that in healthy individuals. In addition, the amount of AGR2 was significantly higher in patients with metastasis. Interestingly, extracellular AGR2 is not only clinically relevant in human tumors, but also significantly correlated with malignant mammary tumor (MMT) progression (P = 0.0007), distant tumor metastasis (P = 0.002) and poor overall survival (P = 0.0158) in dogs (83). In conclusion, we emphasize that AGR2 can be found in the body fluid of cancer patients, and its expression level can be distinguished from that of normal patients, which means that AGR2 may be used as a cancer marker for diagnosis or prognosis. It has certain reference value to infer whether the patient has a primary tumor and whether the tumor has metastasized based on the expression level of AGR2. At the same time, the combination of AGR2 and other biomarkers may be a promising strategy to improve the accuracy of early breast cancer detection (84). However, since AGR2 is not specific to breast, it cannot be used alone for early cancer detection as a serum biomarker, and needs to be integrated into the diagnostic score. So far, the detection of AGR2 protein level by enzyme-linked immunosorbent assay (ELISA) and the detection of AGR2 mRNA level by RT-PCR have extensive practical basis in the clinical detection of AGR2 (15, 85). However, these methods have expensive and complex equipment, so we are looking forward to developing simple, efficient and sensitive methods for detecting AGR2. Aptamers are small fragments of nucleotides or protein peptides that are designed to bind to target molecules with specificity and high affinity (40). Multiple peptide aptamers are screened out by using the combined phage peptide library, which can recognize some epitopes of AGR2. The microarray composed of these peptide aptamers can be used to quantify AGR2 in clinical samples, providing a new and effective method for the determination of clinical markers. Hu et al. (86) showed a simple optical aptamer sensor for detecting AGR2 protein based on gold nanoparticles (AuNPs) and magnetic separation. The designed aptamer sensor is effective, sensitive and has low detection limit, which was successfully completed by using ultraviolet-visible molecular absorption spectrometry (UV-Vis). Lan et al. (87) used AGR aptamer coupled with a cytosine base sequence as Ag cluster template (MA@AgNCs) for targeting intracellular AGR. MA@AgNCs shows the maximum fluorescence peak at 565 and has excellent quantum yield (QY= 87.43%), small size, good biocompatibility, low toxicity and good stability. In addition, synthetic MA@AgNCs shows a high specificity in recognizing breast cancer cells. Graham et al. (88) designed a porous silicon based (PSi) aptamer that detected AGR2 by real-time monitoring the reflectivity changes of PSi nanostructures, with high selectivity and sufficient sensitivity. The emergence of aptamers provides a new research platform for efficient and rapid identification of AGR2, showing a good application prospect.

## AGR2-related drug resistance in breast cancer

The incidence of ER positive breast cancer is the highest, accounting for about 75% of all cases of breast cancer (89). ER positive breast cancer usually responds well to endocrine therapy (ET). Endocrine therapy inhibits estrogen signal transduction in cancer cells, prevents their proliferation (cell inhibitory effect) and induces cell apoptosis (cell killing effect) (90). Although most ER positive breast cancer patients initially respond well to endocrine therapy, drug resistance will develop over time (acquired resistance), or some patients will not respond to endocrine therapy from the beginning (new resistance) (91). Therefore, ET resistance is an important clinical challenge in the treatment of breast cancer. Clinical studies (5, 92) had shown that the increased expression of AGR2 could mediate the resistance of tamoxifen as an estrogen agonist. Therefore, AGR2 level can be used to predict the resistance to tamoxifen and poor treatment response. Hrstka et al. (93) made tumor cells sensitive to tamoxifen by inhibiting the PDPK1-AKT pathway, which helped to exhaust the level of AGR2 protein, confirming the above view. Zamzam et al. (94) divided 224 ER positive breast cancer patients into three groups. Group 1 was sensitive to tamoxifen. Group 2 and group 3 were resistant to tamoxifen, and the level of AGR2 protein in all patients was mainly detected by ELISA. After 5 years of follow-up, they found that compared with group 1, the serum AGR2 level in group 2 and group 3 was significantly increased. This indicated that although ER expression itself was the main predictor of endocrine therapy response, the expression of AGR2 was closely related to the resistance of ER positive breast cancer patients to endocrine drugs (95), and serum AGR2 has potential availability as a biomarker for noninvasive early detection of tamoxifen resistance by ELISA. In addition to tamoxifen, Li et al. (48) found that the level of endogenous AGR2 was positively correlated with the resistance to fulvestrant in MCF-7 and T47D cells. The knockdown of AGR2 in MCF-7 cells strongly enhanced the G1 phase arrest and accelerated the degradation of ERα induced by fulvestrant. They also found that fulvestrant not only induced ER to enter the nucleus, but also caused AGR2 to relocate to the outer edge of the cell. This might be due to the conformational changes induced by fulvestrant and the subsequent phosphorylation of endoplasmic reticulum releasing AGR2 bound to ER. After treatment with fulvestrant, most of the ER entered the nucleus and released the bound AGR2. In addition, AGR2 can also communicate with HIF-1α, leading to hypoxia induced adriamycin resistance (96). The knockdown of AGR2 in MCF-7 cells led to the inhibition of adriamycin resistance induced by HIF-1α, while the increase of AGR2 level in MDA-MB-231 cells could enhance adriamycin resistance. A methyltransferase METTL3 may modify MALAT1 protein through N6 methyladenosine (m6A), recruit E2F1 and activate the expression of downstream AGR2, thus promoting the adriamycin resistance in breast cancer (97). Maarouf et al. (73) observed that the expression of AGR2 in tumor was negatively correlated with the aging marker p16. AGR2 induced the reproliferation of aging cells by activating AKT and mTORC2 signal transduction, leading to chemotherapy resistance. Whether it is by stabilizing HIF-1α to mediate the multiple drug resistance of breast cancer cells (96), or by promoting the localization of EGFR in the cell membrane, enhancing the EGFR signal to cause cancer cell proliferation (31), or by inhibiting the cell survival p38 MAPK pathway, inhibiting the activation of p53 transcription, and increasing the drug resistance of tumor cells to DNA damage drugs (43), it shows that the overexpression of AGR2 plays an important role in the treatment of breast cancer resistance.

## Potential therapeutic targets in breast cancer

With the gradual deepening of research, the key regulation of AGR2 in cancer is gradually clear. It can be used as a cytoplasmic protein or through secretory form, mediate inflammatory response and external stimuli, regulate endoplasmic reticulum stress, affect the activity of p53, so as to regulate the survival, adhesion and metastasis of tumor cells, enhance the malignant transformation of tumor and promote the resistance of cancer cells to drugs. Therefore, AGR2 is an important target for cancer treatment.

The first antibody developed against AGR2 is a mouse monoclonal antibody, called 18A4, which has been proved to inhibit the growth of breast cancer cells *in vitro (* 98). Subsequent studies produced a humanized version of this antibody, 18A4Hu I, and reported that it inhibited the growth of AGR2 positive ovarian cancer xenografts (99). The AGR2 monoclonal antibody aims to specifically target the extracellular AGR2, without affecting the intracellular AGR2 retention protein associated with endoplasmic reticulum (100). Recently, in a preclinical mouse model of lung cancer, 18A4 antibody has been shown to improve survival and prevent AGR2 induced tumor progression by regulating p53 and MAPK pathways, without any toxic effect on major organs (101, 102). A study by Cocce et al. (103) based on the transcription factor FOXA1, membrane receptor LYPD3 and its ligand AGR2, identified a new target pathway for endocrine therapy of drug-resistant breast cancer. They showed that inhibiting the activity of this pathway with blocking antibodies against LYPD3 or AGR2 inhibited the growth of endocrine therapy resistant breast cancer in the preclinical model, and again provided the basis for the development of humanized antibodies against AGR2. Jung et al. (51) found that Twist1 is a new transcription factor that controls the expression of AGR2, and AGR2 is a key factor in Twist1 mediated breast cancer cell proliferation and migration. Therefore, targeting ER and Twist1 pathway at the same time may be enough to inhibit AGR2 and improve the survival rate of breast cancer patients. Considering the difference of AGR2 expression levels in different breast cancer patients, Zhang et al. (104) divided samples from patients with breast cancer into the high and low AGR2 expression subgroups. They found that patients with relatively AGR2 low expression exhibited immune “hot” tumors and immunosuppressive phenotype with high abundance of tumor immune cell infiltration, while patients with AGR2 high expression displayed opposite immunological characteristics, lacking immune cell infiltration. The outcome suggests that breast cancer patients with relatively AGR2 low expression may be more suitable for the treatment of immunotherapy, while the AGR2 high expression subgroup can firstly inhibit the expression of AGR2 by monoclonal antibody and transform poor immunogenic (cold) tumors into highly immunogenic and well-infiltrated (hot) tumors, which provides a personalized immunotherapy strategy for breast cancer based on AGR2. At the same time, bispecific antibodies (BsAb) have gradually become popular. In breast cancer, although PD-1/PD-L1 inhibitors have been proved to be more effective and less toxic than chemotherapy, immune related adverse events (irAE) have been observed, and in some cases, they may be related to irreversible organ damage or death (105). If AGR2 antibody and PD-1/PD-L1 inhibitors are combined to form a bispecific antibody, taking advantage of the increased expression of AGR2 in tumor cells, AGR2 antibody targets the tumor microenvironment and guides PD-1/PD-L1 inhibitors to enrich in the tumor bodies, thus reducing the non-specific over-activation of the immune system and maintaining the original or even additional tumor killing effect. Roy et al. (106) designed and synthesized BsAb AGR2xPD1, which showed higher anti-tumor response compared with the group of 18A4HU monoclonal antibody (mAb), the group of PD1 mAb and the combination treatment group of 18A4HU mAb and PD1 mAb. Wang et al. (107) focused their research on inhibiting AGR2 expression on proteasome inhibitors. They found that proteasome inhibitor MG132/bortezomib inhibited AGR2 expression at both mRNA and protein levels by activating autophagy. The combination of proteasome inhibitor and bevacizumab could enhance the anti-tumor efficiency of bevacizumab by decreasing the expression level of AGR2 and reducing its role in tumor cell angiogenesis. However, autophagy plays a dual role in tumor cell survival during chemotherapy and cancer gene targeting therapy, which means that cells can also recycle organelles to provide an energy supply by activating autophagy, leading to drug resistance (108). Therefore, more studies are needed in the future to prove the potential inhibitory effect of proteasome inhibitors alone or in combination with targeted drugs on the growth and metastasis of breast cancer and the benefits of clinical transformation. In addition to ER positive breast cancer, in HER2 positive breast cancer, ER signal transduction may also act as an escape pathway (109), leading to resistance to HER2 therapy. Therefore, blocking AGR2 directly may be an option for patients with HER2 positive breast tumors (52). The development and application of AGR2 targeted monoclonal antibodies, selective peptides and microRNAs can inhibit the growth and migration of breast cancer cells and enhance drug sensitivity (110). Zhang et al. (111) designed a hexapeptide based on the combination of AGR2 with the largest subunit of RNA Polymerase II (RNAPII) in a peptide motif dependent manner, which interfered with RNAPII by competitively destroying the AGR2-RNAPII complex, leading to RNAPII dysfunction and accompanied by the activation of DNA damage response in early tumor lesions, and proved to be effective in the treatment of breast cancer. It is worth mentioning that because the key linear motif of AGR2 protein exists in CXXS motif rather than in CXXC motif, AGR2 protein is more likely to form a homodimer to attain the same redox capacity. The stability of the dimer can be changed by studying a drug precursor to mediate the disorder region at the N-terminal of the protein, thus affecting the function of AGR2 in breast cancer. In the near future, it has a good prospect to test and apply AGR2 antibody in clinical trials and clinical patients.

## Conclusion

In the past few years, AGR2 protein has aroused great interest in oncology. Its various carcinogenic properties and pathological effects mainly depend on the specificity of its cellular or extracellular localization. Intracellular AGR2 is a catalyst for the protein balance of endoplasmic reticulum to meet the secretory needs of cancer cells, while extracellular AGR2 is involved in the pro cancer signal transduction of epithelial tumorigenesis, ECM remodeling, inflammatory response and angiogenesis. In addition, this secreted AGR2 can be found in the body fluid of cancer patients, and the expression level can be distinguished from normal patients, which indicates that AGR2 can be used as a marker for diagnosis, prognosis and drug resistance. Diagnostic tools such as microfluidic detection devices or biosensors can be developed to detect AGR2 specifically and sensitively. Combining AGR2 with other tumor markers can improve the sensitivity of breast cancer diagnosis, which is one of the hot spots that clinicians need to pay attention to in the future. So far, therapeutic strategies targeting AGR2 have shown promising results. For example, by constructing the bispecific antibodies of AGR2 antibody and immune checkpoint proteins, it can play its role in tumor tissue with maximum target concentration, which is a clinical transformation direction to improve the efficacy and reduce side effects. However, we also need to study the changes of key genes in AGR2 related signaling pathways, and better understand the upstream and downstream molecular mechanisms of AGR2. The in-depth understanding of the mechanism of AGR2 is of great significance for the study of the mechanism of tumor occurrence and development, as well as the early diagnosis, treatment and prognosis of AGR2 as a molecular target in clinic.

## Author contributions

KZ: investigation, data curation, visualization, methodology, and writing-original draft. YL: investigation, visualization, writing-original draft, and funding acquisition. XK: methodology, writing-original draft, and writing-review and editing. CL: writing-original draft, and funding acquisition. HY: writing-original draft, and funding acquisition. NW: data curation and writing-original draft. ZW: conceptualization, supervision, validation, and project administration. HC: validation, supervision, and writing-review and editing. LX: conceptualization, resources, supervision, and funding acquisition. All authors contributed to the article and approved the submitted version.

## References

[1] Thompson DA Weigel RJ. Hag-2, the human homologue of the xenopus laevis cement gland gene xag-2, is coexpressed with estrogen receptor in breast cancer cell lines. Biochem Biophys Res Commun (1998) 251(1):111–6. doi: 10.1006/bbrc.1998.9440 9790916

[2] Salmans ML Zhao F Andersen B. The estrogen-regulated anterior gradient 2 (Agr2) protein in breast cancer: a potential drug target and biomarker. Breast Cancer Res (2013) 15(2):204. doi: 10.1186/bcr3408 23635006 PMC3672732

[3] Petek E Windpassinger C Egger H Kroisel PM Wagner K. Localization of the human anterior gradient-2 gene (Agr2) to chromosome band 7p21.3 by radiation hybrid mapping and fluorescencein situ hybridisation. Cytogenet Cell Genet (2000) 89(3-4):141–2. doi: 10.1159/000015594 10965104

[4] Shih LJ Lu YF Chen YH Lin CC Chen JA Hwang SP. Characterization of the Agr2 gene, a homologue of X. Laevis Anterior Gradient 2 Zebrafish Danio Rerio. Gene Expr Patterns (2007) 7(4):452–60. doi: 10.1016/j.modgep.2006.11.003 17175205

[5] Hrstka R Nenutil R Fourtouna A Maslon MM Naughton C Langdon S. The pro-metastatic protein anterior gradient-2 predicts poor prognosis in tamoxifen-treated breast cancers. Oncogene (2010) 29(34):4838–47. doi: 10.1038/onc.2010.228 20531310

[6] Persson S Rosenquist M Knoblach B Khosravi-Far R Sommarin M Michalak M. Diversity of the protein disulfide isomerase family: identification of breast tumor induced Hag2 and Hag3 as novel members of the protein family. Mol Phylogenet Evol (2005) 36(3):734–40. doi: 10.1016/j.ympev.2005.04.002 15935701

[7] Shishkin SS Eremina LS Kovalev LI Kovaleva MA. Agr2, Erp57/Grp58, and some other human protein disulfide isomerases. Biochem (Mosc) (2013) 78(13):1415–30. doi: 10.1134/S000629791313004X 24490732

[8] Park SW Zhen G Verhaeghe C Nakagami Y Nguyenvu LT Barczak AJ. The protein disulfide isomerase Agr2 is essential for production of intestinal mucus. Proc Natl Acad Sci U.S.A. (2009) 106(17):6950–5. doi: 10.1073/pnas.0808722106 PMC2678445 19359471

[9] Moidu NA AR NS Syafruddin SE Low TY Mohtar MA. Secretion of pro-oncogenic Agr2 protein in cancer. Heliyon (2020) 6(9):e05000. doi: 10.1016/j.heliyon.2020.e05000 33005802 PMC7519367

[10] Brychtova V Mohtar A Vojtesek B Hupp TR. Mechanisms of anterior gradient-2 regulation and function in cancer. Semin Cancer Biol (2015) 33:16–24. doi: 10.1016/j.semcancer.2015.04.005 25937245

[11] Gupta A Dong A Lowe AW. Agr2 gene function requires a unique endoplasmic reticulum localization motif. J Biol Chem (2012) 287(7):4773–82. doi: 10.1074/jbc.M111.301531 PMC3281655 22184114

[12] Raykhel I Alanen H Salo K Jurvansuu J Nguyen VD Latva-Ranta M. A molecular specificity code for the three mammalian kdel receptors. J Cell Biol (2007) 179(6):1193–204. doi: 10.1083/jcb.200705180 PMC2140024 18086916

[13] Chevet E Fessart D Delom F Mulot A Vojtesek B Hrstka R. Emerging roles for the pro-oncogenic anterior gradient-2 in cancer development. Oncogene (2013) 32(20):2499–509. doi: 10.1038/onc.2012.346 22945652

[14] Dumartin L Whiteman HJ Weeks ME Hariharan D Dmitrovic B Iacobuzio-Donahue CA. Agr2 is a novel surface antigen that promotes the dissemination of pancreatic cancer cells through regulation of cathepsins b and d. Cancer Res (2011) 71(22):7091–102. doi: 10.1158/0008-5472.CAN-11-1367 PMC3541941 21948970

[15] Shi T Gao Y Quek SI Fillmore TL Nicora CD Su D. A highly sensitive targeted mass spectrometric assay for quantification of Agr2 protein in human urine and serum. J Proteome Res (2014) 13(2):875–82. doi: 10.1021/pr400912c PMC3975687 24251762

[16] Park K Chung YJ So H Kim K Park J Oh M. Agr2, a mucinous ovarian cancer marker, promotes cell proliferation and migration. Exp Mol Med (2011) 43(2):91–100. doi: 10.3858/emm.2011.43.2.011 21200134 PMC3047197

[17] Fessart D Domblides C Avril T Eriksson LA Begueret H Pineau R. Secretion of protein disulphide isomerase Agr2 confers tumorigenic properties. Elife (2016) 5:e13887. doi: 10.7554/eLife.13887 27240165 PMC4940162

[18] Zhang H Chi J Hu J Ji T Luo Z Zhou C. Intracellular Agr2 transduces Pge2 stimuli to promote epithelial-mesenchymal transition and metastasis of colorectal cancer. Cancer Lett (2021) 518:180–95. doi: 10.1016/j.canlet.2021.06.025 34216690

[19] Jessop CE Watkins RH Simmons JJ Tasab M Bulleid NJ. Protein disulphide isomerase family members show distinct substrate specificity: P5 is targeted to bip client proteins. J Cell Sci (2009) 122(Pt 23):4287–95. doi: 10.1242/jcs.059154 PMC2779130 19887585

[20] Pohler E Craig AL Cotton J Lawrie L Dillon JF Ross P. The barrett's antigen anterior gradient-2 silences the P53 transcriptional response to DNA damage. Mol Cell Proteomics (2004) 3(6):534–47. doi: 10.1074/mcp.M300089-MCP200 14967811

[21] Norris AM Gore A Balboni A Young A Longnecker DS Korc M. Agr2 is a Smad4-suppressible gene that modulates Muc1 levels and promotes the initiation and progression of pancreatic intraepithelial neoplasia. Oncogene (2013) 32(33):3867–76. doi: 10.1038/onc.2012.394 PMC3515713 22945649

[22] Schroeder BW Verhaeghe C Park SW Nguyenvu LT Huang X Zhen G. Agr2 is induced in asthma and promotes allergen-induced mucin overproduction. Am J Respir Cell Mol Biol (2012) 47(2):178–85. doi: 10.1165/rcmb.2011-0421OC PMC3423459 22403803

[23] Patel P Clarke C Barraclough DL Jowitt TA Rudland PS Barraclough R. Metastasis-promoting anterior gradient 2 protein has a dimeric thioredoxin fold structure and a role in cell adhesion. J Mol Biol (2013) 425(5):929–43. doi: 10.1016/j.jmb.2012.12.009 23274113

[24] Mohtar MA Hernychova L O'Neill JR Lawrence ML Murray E Vojtesek B. The sequence-specific peptide-binding activity of the protein sulfide isomerase Agr2 directs its stable binding to the oncogenic receptor epcam. Mol Cell Proteomics (2018) 17(4):737–63. doi: 10.1074/mcp.RA118.000573 PMC5880107 29339412

[25] Ryu J Park SG Lee PY Cho S Lee DH Kim GH. Dimerization of pro-oncogenic protein anterior gradient 2 is required for the interaction with Bip/Grp78. Biochem Biophys Res Commun (2013) 430(2):610–5. doi: 10.1016/j.bbrc.2012.11.105 23220234

[26] Bouchalova P Sommerova L Potesil D Martisova A Lapcik P Koci V. Characterization of the Agr2 interactome uncovers new players of protein disulfide isomerase network in cancer cells. Mol Cell Proteomics (2022) 21(2):100188. doi: 10.1016/j.mcpro.2021.100188 34929376 PMC8816719

[27] Ryan DP Matthews JM. Protein-protein interactions in human disease. Curr Opin Struct Biol (2005) 15(4):441–6. doi: 10.1016/j.sbi.2005.06.001 15993577

[28] Delom F Mohtar MA Hupp T Fessart D. The anterior gradient-2 interactome. Am J Physiol Cell Physiol (2020) 318(1):C40–C7. doi: 10.1152/ajpcell.00532.2018 31644305

[29] Maurel M Obacz J Avril T Ding YP Papadodima O Treton X. Control of anterior gradient 2 (Agr2) dimerization links endoplasmic reticulum proteostasis to inflammation. EMBO Mol Med (2019) 11(6):e10120. doi: 10.15252/emmm.201810120 31040128 PMC6554669

[30] Fletcher GC Patel S Tyson K Adam PJ Schenker M Loader JA. Hag-2 and hag-3, human homologues of genes involved in differentiation, are associated with oestrogen receptor-positive breast tumours and interact with metastasis gene C4.4a and dystroglycan. Br J Cancer (2003) 88(4):579–85. doi: 10.1038/sj.bjc.6600740 PMC2377166 12592373

[31] Dong A Wodziak D Lowe AW. Epidermal growth factor receptor (Egfr) signaling requires a specific endoplasmic reticulum thioredoxin for the post-translational control of receptor presentation to the cell surface. J Biol Chem (2015) 290(13):8016–27. doi: 10.1074/jbc.M114.623207 PMC4375459 25666625

[32] Arumugam T Deng D Bover L Wang H Logsdon CD Ramachandran V. New blocking antibodies against novel Agr2-C4.4a pathway reduce growth and metastasis of pancreatic tumors and increase survival in mice. Mol Cancer Ther (2015) 14(4):941–51. doi: 10.1158/1535-7163.MCT-14-0470 PMC4710371 25646014

[33] Maslon MM Hrstka R Vojtesek B Hupp TR. A divergent substrate-binding loop within the pro-oncogenic protein anterior gradient-2 forms a docking site for reptin. J Mol Biol (2010) 404(3):418–38. doi: 10.1016/j.jmb.2010.09.035 20888340

[34] Gray TA Alsamman K Murray E Sims AH Hupp TR. Engineering a synthetic cell panel to identify signalling components reprogrammed by the cell growth regulator anterior gradient-2. Mol Biosyst (2014) 10(6):1409–25. doi: 10.1039/c4mb00113c 24710632

[35] Yu H Zhao J Lin L Zhang Y Zhong F Liu Y. Proteomic study explores Agr2 as pro-metastatic protein in hcc. Mol Biosyst (2012) 8(10):2710–8. doi: 10.1039/c2mb25160d 22828706

[36] Liu Z Hu Y Gong Y Zhang W Liu C Wang Q. Hydrogen peroxide mediated mitochondrial Ung1-Prdx3 interaction and Ung1 degradation. Free Radic Biol Med (2016) 99:54–62. doi: 10.1016/j.freeradbiomed.2016.07.030 27480846

[37] Paret C Bourouba M Beer A Miyazaki K Schnolzer M Fiedler S. Ly6 family member C4.4a binds laminins 1 and 5, associates with galectin-3 and supports cell migration. Int J Cancer (2005) 115(5):724–33. doi: 10.1002/ijc.20977 15729693

[38] Kumar A Godwin JW Gates PB Garza-Garcia AA Brockes JP. Molecular basis for the nerve dependence of limb regeneration in an adult vertebrate. Science (2007) 318(5851):772–7. doi: 10.1126/science.1147710 PMC2696928 17975060

[39] Garza-Garcia A Harris R Esposito D Gates PB Driscoll PC. Solution structure and phylogenetics of Prod1, a member of the three-finger protein superfamily implicated in salamander limb regeneration. PloS One (2009) 4(9):e7123. doi: 10.1371/journal.pone.0007123 19771161 PMC2740830

[40] Murray E McKenna EO Burch LR Dillon J Langridge-Smith P Kolch W. Microarray-formatted clinical biomarker assay development using peptide aptamers to anterior gradient-2. Biochemistry (2007) 46(48):13742–51. doi: 10.1021/bi7008739 17994709

[41] Guo H Zhu Q Yu X Merugu SB Mangukiya HB Smith N. Tumor-secreted anterior gradient-2 binds to vegf and Fgf2 and enhances their activities by promoting their homodimerization. Oncogene (2017) 36(36):5098–109. doi: 10.1038/onc.2017.132 28481872

[42] Gong W Ekmu B Wang X Lu Y Wan L. Agr2-induced glucose metabolism facilitated the progression of endometrial carcinoma Via enhancing the Muc1/Hif-1alpha pathway. Hum Cell (2020) 33(3):790–800. doi: 10.1007/s13577-020-00356-4 32304027

[43] Hrstka R Bouchalova P Michalova E Matoulkova E Muller P Coates PJ. Agr2 oncoprotein inhibits P38 mapk and P53 activation through a Dusp10-mediated regulatory pathway. Mol Oncol (2016) 10(5):652–62. doi: 10.1016/j.molonc.2015.12.003 PMC5423154 26733232

[44] Zhou X Zhang W Dou M Li Z Liu Z Li J. (125)I seeds inhibit proliferation and promote apoptosis in cholangiocarcinoma cells by regulating the Agr2-mediated P38 mapk pathway. Cancer Lett (2022) 524:29–41. doi: 10.1016/j.canlet.2021.10.014 34656689

[45] Zweitzig DR Smirnov DA Connelly MC Terstappen LW O'Hara SM Moran E. Physiological stress induces the metastasis marker Agr2 in breast cancer cells. Mol Cell Biochem (2007) 306(1-2):255–60. doi: 10.1007/s11010-007-9562-y 17694278

[46] Tiemann K Garri C Lee SB Malihi PD Park M Alvarez RM. Loss of er retention motif of Agr2 can impact mtorc signaling and promote cancer metastasis. Oncogene (2019) 38(16):3003–18. doi: 10.1038/s41388-018-0638-9 PMC7523706 30575818

[47] Sommerova L Ondrouskova E Vojtesek B Hrstka R. Suppression of Agr2 in a tgf-Beta-Induced smad regulatory pathway mediates epithelial-mesenchymal transition. BMC Cancer (2017) 17(1):546. doi: 10.1186/s12885-017-3537-5 28810836 PMC5557473

[48] Li Z Wu Z Chen H Zhu Q Gao G Hu L. Induction of anterior gradient 2 (Agr2) plays a key role in insulin-like growth factor-1 (Igf-1)-Induced breast cancer cell proliferation and migration. Med Oncol (2015) 32(6):577. doi: 10.1007/s12032-015-0577-z 25956506 PMC4451465

[49] Hetz C. The unfolded protein response: controlling cell fate decisions under er stress and beyond. Nat Rev Mol Cell Biol (2012) 13(2):89–102. doi: 10.1038/nrm3270 22251901

[50] Dumartin L Alrawashdeh W Trabulo SM Radon TP Steiger K Feakins RM. Er stress protein Agr2 precedes and is involved in the regulation of pancreatic cancer initiation. Oncogene (2017) 36(22):3094–103. doi: 10.1038/onc.2016.459 PMC5467015 27941872

[51] Jung SY Yun J Kim SJ Kang S Kim DY Kim YJ. Basic helix-Loop-Helix transcription factor Twist1 is a novel regulator of anterior gradient protein 2 homolog (Agr2) in breast cancer. Biochem Biophys Res Commun (2019) 516(1):149–56. doi: 10.1016/j.bbrc.2019.05.191 31202462

[52] Ondrouskova E Sommerova L Nenutil R Coufal O Bouchal P Vojtesek B. Agr2 associates with Her2 expression predicting poor outcome in subset of estrogen receptor negative breast cancer patients. Exp Mol Pathol (2017) 102(2):280–3. doi: 10.1016/j.yexmp.2017.02.016 28238761

[53] Xiu B Chi Y Liu L Chi W Zhang Q Chen J. Linc02273 drives breast cancer metastasis by epigenetically increasing Agr2 transcription. Mol Cancer (2019) 18(1):187. doi: 10.1186/s12943-019-1115-y 31856843 PMC6921600

[54] Zhang Y Xia F Zhang F Cui Y Wang Q Liu H. Mir-135b-5p enhances doxorubicin-sensitivity of breast cancer cells through targeting anterior gradient 2. J Exp Clin Cancer Res (2019) 38(1):26. doi: 10.1186/s13046-019-1024-3 30665445 PMC6341729

[55] Wang J Huang K Shi L Zhang Q Zhang S. Circpvt1 promoted the progression of breast cancer by regulating mir-29a-3p-Mediated Agr2-Hif-1alpha pathway. Cancer Manag Res (2020) 12:11477–90. doi: 10.2147/CMAR.S265579 PMC7672658 33223849

[56] Zuo T Jiang P Fu J Zhang Y. Lncrna Afap1-As1 induces gefitinib resistance of lung adenocarcinoma through the mir-653-5p/Agr2 axis. Ther Clin Risk Manag (2023) 19:1–13. doi: 10.2147/TCRM.S374162 36636455 PMC9829986

[57] Tsai HW Chen YL Wang CI Hsieh CC Lin YH Chu PM. Anterior gradient 2 induces resistance to sorafenib Via endoplasmic reticulum stress regulation in hepatocellular carcinoma. Cancer Cell Int (2023) 23(1):42. doi: 10.1186/s12935-023-02879-w 36899352 PMC9999520

[58] Alasiri G Fan LY Zona S Goldsbrough IG Ke HL Auner HW. Er stress and cancer: the foxo forkhead transcription factor link. Mol Cell Endocrinol (2018) 462(Pt B):67–81. doi: 10.1016/j.mce.2017.05.027 28572047

[59] Higa A Mulot A Delom F Bouchecareilh M Nguyen DT Boismenu D. Role of pro-oncogenic protein disulfide isomerase (Pdi) family member anterior gradient 2 (Agr2) in the control of endoplasmic reticulum homeostasis. J Biol Chem (2011) 286(52):44855–68. doi: 10.1074/jbc.M111.275529 PMC3248018 22025610

[60] Martisova A Sommerova L Krejci A Selingerova I Kolarova T Zavadil Kokas F. Identification of Agr2 gene-specific expression patterns associated with epithelial-mesenchymal transition. Int J Mol Sci (2022) 23(18):10845. doi: 10.3390/ijms231810845 36142758 PMC9504245

[61] Vitello EA Quek SI Kincaid H Fuchs T Crichton DJ Troisch P. Cancer-secreted Agr2 induces programmed cell death in normal cells. Oncotarget (2016) 7(31):49425–34. doi: 10.18632/oncotarget.9921 PMC5226518 27283903

[62] Clarke C Rudland P Barraclough R. The metastasis-inducing protein Agr2 is O-glycosylated upon secretion from mammary epithelial cells. Mol Cell Biochem (2015) 408(1-2):245–52. doi: 10.1007/s11010-015-2502-3 PMC4768226 26169982

[63] Jia M Guo Y Zhu D Zhang N Li L Jiang J. Pro-metastatic activity of Agr2 interrupts angiogenesis target bevacizumab efficiency via direct interaction with vegfa and activation of nf-kappab pathway. Biochim Biophys Acta Mol Basis Dis (2018) 1864(5 Pt A):1622–33. doi: 10.1016/j.bbadis.2018.01.021 29410027

[64] Merugu SB Zhou B Mangukiya HB Negi H Ghulam R Roy D. Extracellular Agr2 activates neighboring fibroblasts through endocytosis and direct binding to beta-catenin that requires Agr2 dimerization and adhesion domains. Biochem Biophys Res Commun (2021) 573:86–92. doi: 10.1016/j.bbrc.2021.08.028 34399098

[65] Zhu Q Mangukiya HB Mashausi DS Guo H Negi H Merugu SB. Anterior gradient 2 is induced in cutaneous wound and promotes wound healing through its adhesion domain. FEBS J (2017) 284(17):2856–69. doi: 10.1111/febs.14155 28665039

[66] Kurpinska A Suraj-Prazmowska J Stojak M Jarosz J Mateuszuk L Niedzielska-Andres E. Comparison of anti-cancer effects of novel protein disulphide isomerase (Pdi) inhibitors in breast cancer cells characterized by high and low Pdia17 expression. Cancer Cell Int (2022) 22(1):218. doi: 10.1186/s12935-022-02631-w 35725466 PMC9208212

[67] Liu D Rudland PS Sibson DR Platt-Higgins A Barraclough R. Human homologue of cement gland protein, a novel metastasis inducer associated with breast carcinomas. Cancer Res (2005) 65(9):3796–805. doi: 10.1158/0008-5472.CAN-04-3823 15867376

[68] Innes HE Liu D Barraclough R Davies MP O'Neill PA Platt-Higgins A. Significance of the metastasis-inducing protein Agr2 for outcome in hormonally treated breast cancer patients. Br J Cancer (2006) 94(7):1057–65. doi: 10.1038/sj.bjc.6603065 PMC2361240 16598187

[69] Fritzsche FR Dahl E Pahl S Burkhardt M Luo J Mayordomo E. Prognostic relevance of Agr2 expression in breast cancer. Clin Cancer Res (2006) 12(6):1728–34. doi: 10.1158/1078-0432.CCR-05-2057 16551856

[70] Barraclough DL Platt-Higgins A de Silva Rudland S Barraclough R Winstanley J West CR. The metastasis-associated anterior gradient 2 protein is correlated with poor survival of breast cancer patients. Am J Pathol (2009) 175(5):1848–57. doi: 10.2353/ajpath.2009.090246 PMC2774050 19834055

[71] Ann P Seagle BL Shilpi A Kandpal M Shahabi S. Association of increased primary breast tumor Agr2 with decreased disease-specific survival. Oncotarget (2018) 9(33):23114–25. doi: 10.18632/oncotarget.25225 PMC5955412 29796176

[72] Kereh DS Pieter J Hamdani W Haryasena H Sampepajung D Prihantono P. Correlation of Agr2 expression with the incidence of metastasis in luminal breast cancer. Breast Dis (2021) 40(S1):S103–S7. doi: 10.3233/BD-219015 34092584

[73] Maarouf A Boissard A Henry C Leman G Coqueret O Guette C. Anterior gradient protein 2 is a marker of tumor aggressiveness in breast cancer and favors Chemotherapy−Induced senescence escape. Int J Oncol (2022) 60(1):5. doi: 10.3892/ijo.2021.5295 34913074 PMC8727137

[74] Lacambra MD Tsang JY Ni YB Chan SK Tan PH Tse GM. Anterior gradient 2 is a poor outcome indicator in luminal breast cancer. Ann Surg Oncol (2015) 22(11):3489–96. doi: 10.1245/s10434-015-4420-8 25663596

[75] Wilson CL Sims AH Howell A Miller CJ Clarke RB. Effects of oestrogen on gene expression in epithelium and stroma of normal human breast tissue. Endocr Relat Cancer (2006) 13(2):617–28. doi: 10.1677/erc.1.01165 16728587

[76] Vanderlaag KE Hudak S Bald L Fayadat-Dilman L Sathe M Grein J. Anterior gradient-2 plays a critical role in breast cancer cell growth and survival by modulating cyclin D1, estrogen receptor-alpha and survivin. Breast Cancer Res (2010) 12(3):R32. doi: 10.1186/bcr2586 20525379 PMC2917027

[77] de Moraes CL Cruz EMN Valoyes MAV Naves do Amaral W. Agr2 and Agr3 play an important role in the clinical characterization and prognosis of basal like breast cancer. Clin Breast Cancer (2022) 22(2):e242–e52. doi: 10.1016/j.clbc.2021.07.008 34462207

[78] Wang J Xu B. Targeted therapeutic options and future perspectives for Her2-positive breast cancer. Signal Transduct Target Ther (2019) 4:34. doi: 10.1038/s41392-019-0069-2 31637013 PMC6799843

[79] Duran MC Vega F Moreno-Bueno G Artiga MJ Sanchez L Palacios J. Characterisation of tumoral markers correlated with Erbb2 (Her2/Neu) overexpression and metastasis in breast cancer. Proteomics Clin Appl (2008) 2(9):1313–26. doi: 10.1002/prca.200780020 21136925

[80] Fessart D Villamor I Chevet E Delom F Robert J. Integrative analysis of genomic and transcriptomic alterations of Agr2 and Agr3 in cancer. Open Biol (2022) 12(7):220068. doi: 10.1098/rsob.220068 35857928 PMC9277299

[81] Jach D Cheng Y Prica F Dumartin L Crnogorac-Jurcevic T. From development to cancer - an ever-increasing role of Agr2. Am J Cancer Res (2021) 11(11):5249–62. PMC8640830 34873459

[82] Tian SB Tao KX Hu J Liu ZB Ding XL Chu YN. The prognostic value of Agr2 expression in solid tumours: a systematic review and meta-analysis. Sci Rep (2017) 7(1):15500. doi: 10.1038/s41598-017-15757-z 29138453 PMC5686151

[83] Yuan SH Chang SC Huang Y Liu HP. Serum level of tumor-overexpressed Agr2 is significantly associated with unfavorable prognosis of canine malignant mammary tumors. Anim (Basel) (2021) 11(10):2923. doi: 10.3390/ani11102923 PMC8532596 34679944

[84] Whitwell HJ Worthington J Blyuss O Gentry-Maharaj A Ryan A Gunu R. Improved early detection of ovarian cancer using longitudinal multimarker models. Br J Cancer (2020) 122(6):847–56. doi: 10.1038/s41416-019-0718-9 PMC7078315 31937926

[85] Wayner EA Quek SI Ahmad R Ho ME Loprieno MA Zhou Y. Development of an Elisa to detect the secreted prostate cancer biomarker Agr2 in voided urine. Prostate (2012) 72(9):1023–34. doi: 10.1002/pros.21508 22072305

[86] Aydogdu Tig G Pekyardimci S. An electrochemical sandwich-type aptasensor for determination of lipocalin-2 based on graphene Oxide/Polymer composite and gold nanoparticles. Talanta (2020) 210:120666. doi: 10.1016/j.talanta.2019.120666 31987191

[87] Lan J Wu X Luo L Liu J Yang L Wang F. Fluorescent Ag clusters conjugated with anterior gradient-2 antigen aptamer for specific detection of cancer cells. Talanta (2019) 197:86–91. doi: 10.1016/j.talanta.2018.12.089 30771992

[88] Arshavsky-Graham S Ward SJ Massad-Ivanir N Scheper T Weiss SM Segal E. Porous silicon-based aptasensors: toward cancer protein biomarker detection. ACS Meas Sci Au (2021) 1(2):82–94. doi: 10.1021/acsmeasuresciau.1c00019 34693403 PMC8532149

[89] DeSantis CE Ma J Gaudet MM Newman LA Miller KD Goding Sauer A. Breast cancer statistics, 2019. CA Cancer J Clin (2019) 69(6):438–51. doi: 10.3322/caac.21583 31577379

[90] Coser KR Wittner BS Rosenthal NF Collins SC Melas A Smith SL. Antiestrogen-resistant subclones of mcf-7 human breast cancer cells are derived from a common monoclonal drug-resistant progenitor. Proc Natl Acad Sci U.S.A. (2009) 106(34):14536–41. doi: 10.1073/pnas.0907560106 PMC2732824 19706540

[91] Saatci O Huynh-Dam KT Sahin O. Endocrine resistance in breast cancer: from molecular mechanisms to therapeutic strategies. J Mol Med (Berl) (2021) 99(12):1691–710. doi: 10.1007/s00109-021-02136-5 PMC8611518 34623477

[92] Hengel SM Murray E Langdon S Hayward L O'Donoghue J Panchaud A. Data-independent proteomic screen identifies novel tamoxifen agonist that mediates drug resistance. J Proteome Res (2011) 10(10):4567–78. doi: 10.1021/pr2004117 PMC3242698 21936522

[93] Hrstka R Murray E Brychtova V Fabian P Hupp TR Vojtesek B. Identification of an akt-dependent signalling pathway that mediates tamoxifen-dependent induction of the pro-metastatic protein anterior gradient-2. Cancer Lett (2013) 333(2):187–93. doi: 10.1016/j.canlet.2013.01.034 23354592

[94] Zamzam Y Abdelmonem Zamzam Y Aboalsoud M Harras H. The utility of Sox2 and Agr2 biomarkers as early predictors of tamoxifen resistance in er-positive breast cancer patients. Int J Surg Oncol (2021) 2021:9947540. doi: 10.1155/2021/9947540 34567804 PMC8460385

[95] Wright TM Wardell SE Jasper JS Stice JP Safi R Nelson ER. Delineation of a Foxa1/Eralpha/Agr2 regulatory loop that is dysregulated in endocrine therapy-resistant breast cancer. Mol Cancer Res (2014) 12(12):1829–39. doi: 10.1158/1541-7786.MCR-14-0195 PMC4272635 25100862

[96] Li Z Zhu Q Hu L Chen H Wu Z Li D. Anterior gradient 2 is a binding stabilizer of hypoxia inducible factor-1alpha that enhances Cocl2 -induced doxorubicin resistance in breast cancer cells. Cancer Sci (2015) 106(8):1041–9. doi: 10.1111/cas.12714 PMC4556394 26079208

[97] Li S Jiang F Chen F Deng Y Pan X. Effect of M6a methyltransferase Mettl3 -mediated Malat1/E2f1/Agr2 axis on adriamycin resistance in breast cancer. J Biochem Mol Toxicol (2022) 36(1):e22922. doi: 10.1002/jbt.22922 34964205

[98] Wu ZH Zhu Q Gao GW Zhou CC Li DW. [Preparation, characterization and potential application of monoclonal antibody 18a4 against Agr2]. Xi Bao Yu Fen Zi Mian Yi Xue Za Zhi (2010) 26(1):49–51. 20056089

[99] Guo H Chen H Zhu Q Yu X Rong R Merugu SB. A humanized monoclonal antibody targeting secreted anterior gradient 2 effectively inhibits the xenograft tumor growth. Biochem Biophys Res Commun (2016) 475(1):57–63. doi: 10.1016/j.bbrc.2016.05.033 27166154

[100] Boisteau E Posseme C Di Modugno F Edeline J Coulouarn C Hrstka R. Anterior gradient proteins in gastrointestinal cancers: from cell biology to pathophysiology. Oncogene (2022) 41(42):4673–85. doi: 10.1038/s41388-022-02452-1 36068336

[101] Negi H Merugu SB Mangukiya HB Li Z Zhou B Sehar Q. Anterior gradient-2 monoclonal antibody inhibits lung cancer growth and metastasis by upregulating P53 pathway and without exerting any toxicological effects: a preclinical study. Cancer Lett (2019) 449:125–34. doi: 10.1016/j.canlet.2019.01.025 30685412

[102] Liu AY Kanan AD Radon TP Shah S Weeks ME Foster JM. Agr2, a unique tumor-associated antigen, is a promising candidate for antibody targeting. Oncotarget (2019) 10(42):4276–89. doi: 10.18632/oncotarget.26945 PMC6611513 31303962

[103] Cocce KJ Jasper JS Desautels TK Everett L Wardell S Westerling T. The lineage determining factor Grhl2 collaborates with Foxa1 to establish a targetable pathway in endocrine therapy-resistant breast cancer. Cell Rep (2019) 29(4):889–903.e10. doi: 10.1016/j.celrep.2019.09.032 31644911 PMC6874102

[104] Zhang S Liu Q Wei Y Xiong Y Gu Y Huang Y. Anterior gradient-2 regulates cell communication by coordinating cytokine-chemokine signaling and immune infiltration in breast cancer. Cancer Sci (2023) 00:1–16. doi: 10.1111/cas.15775 PMC10236615 36853166

[105] Zhang K Kong X Li Y Wang Z Zhang L Xuan L. Pd-1/Pd-L1 inhibitors in patients with preexisting autoimmune diseases. Front Pharmacol (2022) 13:854967. doi: 10.3389/fphar.2022.854967 35370736 PMC8971753

[106] Roy D Liu GS Zeling Wang A Zhou B Yunus FU Raza G. Construction and stable gene expression of Agr2xpd1 bi-specific antibody that enhances attachment between T-cells and lung tumor cells, suppress tumor cell migration and promoting Cd8 expression in cytotoxic T-cells. Saudi Pharm J (2023) 31(1):85–95. doi: 10.1016/j.jsps.2022.11.007 36685298 PMC9845114

[107] Wang D Xu Q Yuan Q Jia M Niu H Liu X. Proteasome inhibition boosts autophagic degradation of ubiquitinated-Agr2 and enhances the antitumor efficiency of bevacizumab. Oncogene (2019) 38(18):3458–74. doi: 10.1038/s41388-019-0675-z PMC6756021 30647455

[108] Wang X Yu J Liu X Luo D Li Y Song L. Psmg2-controlled proteasome-autophagy balance mediates the tolerance for mek-targeted therapy in triple-negative breast cancer. Cell Rep Med (2022) 3(9):100741. doi: 10.1016/j.xcrm.2022.100741 36099919 PMC9512673

[109] Paplomata E Nahta R O'Regan RM. Systemic therapy for early-stage Her2-positive breast cancers: time for a less-Is-More approach? Cancer (2015) 121(4):517–26. doi: 10.1002/cncr.29060 25346473

[110] Hong X Li ZX Hou J Zhang HY Zhang CY Zhang J. Effects of er-resident and secreted Agr2 on cell proliferation, migration, invasion, and survival in panc-1 pancreatic cancer cells. BMC Cancer (2021) 21(1):33. doi: 10.1186/s12885-020-07743-y 33413231 PMC7791724

[111] Zhang Z Li H Deng Y Schuck K Raulefs S Maeritz N. Agr2-dependent nuclear import of rna polymerase ii constitutes a specific target of pancreatic ductal adenocarcinoma in the context of wild-type P53. Gastroenterology (2021) 161(5):1601–14.e23. doi: 10.1053/j.gastro.2021.07.030 34303658

